# Examining the association between cardiovascular outcomes in individuals undergoing percutaneous coronary intervention with or without a preceding coronary CT angiogram

**DOI:** 10.1093/ehjimp/qyag062

**Published:** 2026-04-17

**Authors:** Khalid Sawalha, Bhupendar Tayal, Kristian Hay Kragholm, Aakash Rana, Brad Fugere, Robert F Spraggins, Landon Bruich, Kedar Jambhekar, Jennifer Rymer, Pamela S Douglas, Subhi J Al’Aref

**Affiliations:** Division of Cardiology, University of Arkansas for Medical Sciences, 4301 West Markham Street, Little Rock, AR 72205, USA; Division of Cardiology, University of Arkansas for Medical Sciences, 4301 West Markham Street, Little Rock, AR 72205, USA; Division of Cardiovascular Disease, Aalborg University Hospital, Aalborg, Denmark; Department of Internal Medicine, Central Arkansas Health Care System, Little Rock, AR, USA; College of Medicine, University of Arkansas for Medical Sciences, Little Rock, AR, USA; Department of Internal Medicine, University of Arkansas for Medical Sciences, Little Rock, AR, USA; Department of Internal Medicine, University of Arkansas for Medical Sciences, Little Rock, AR, USA; Department of Radiology, University of Arkansas for Medical Sciences, Little Rock, AR, USA; Duke Clinical Research Institute, Duke University, Durham, NC, USA; Division of Cardiology, Duke University Medical Center, Durham, NC, USA; Duke Clinical Research Institute, Duke University, Durham, NC, USA; Division of Cardiology, Duke University Medical Center, Durham, NC, USA; Division of Cardiology, University of Arkansas for Medical Sciences, 4301 West Markham Street, Little Rock, AR 72205, USA

**Keywords:** Coronary Computed Tomography Angiography, percutaneous coronary intervention, cardiovascular outcomes

## Abstract

**Aims:**

The role of coronary CT angiography (CCTA) in improving outcomes among patients undergoing percutaneous coronary intervention (PCI) for obstructive coronary artery disease (CAD) remains unclear.

**Methods and results:**

A retrospective cohort study using the TriNetX U.S. Research Network identified adults (≥18 years) who underwent *de novo* PCI (2013–2025). Patients with prior or presenting PCI, coronary artery bypass grafting, or acute coronary syndrome were excluded. Those with CCTA within 1 year before PCI were compared to those without CCTA. After 1:1 propensity score matching, 4936 patients remained in each group. The primary outcome was a composite of all-cause mortality, myocardial infarction (MI), or heart failure (HF) at 1 and 5 years. Baseline characteristics were well-balanced post-matching. CCTA prior to PCI was associated with lower rates of the composite outcome at 1 year (9.6% vs. 13.0%; hazard ratios (HR) 0.74, 95% CI 0.66–0.83) and 5 years (16.1% vs. 23.7%; HR 0.79, 95% CI 0.72–0.86). Reductions were mainly driven by lower MI and mortality rates. HF showed no difference at 1 year but was significantly lower at 5 years (HR 0.85, 95% CI 0.76–0.95).

**Conclusion:**

Pre-procedural CCTA within 1 year of PCI was associated with improved cardiovascular outcomes at 1 and 5 years, suggesting potential benefits from enhanced anatomical assessment and medical optimization before revascularization.

## Graphical Abstract

**Graphical Abstract:**
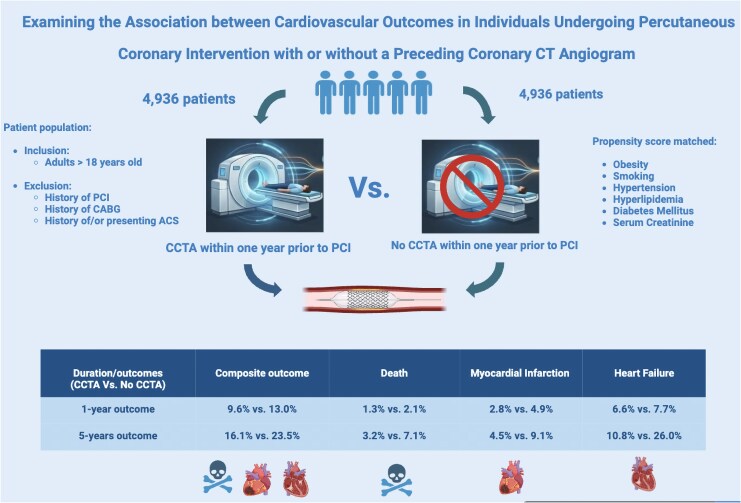

Percutaneous coronary intervention (PCI) performed for the management of stable and unstable coronary artery disease (CAD) has been instrumental in improving short- and long-term outcomes, as well as quality of life indices. However, refining outcomes through enhanced procedural planning as well as pre-procedural medical optimization remains a subject of ongoing investigation.^1^ Coronary CT angiography (CCTA), a noninvasive modality for anatomical and functional assessment of CAD, has demonstrated significant utility in diagnosis, risk stratification, and management decisions specifically for the exclusion of obstructive CAD in intermediate risk cohorts.^1,2^ Furthermore, the use of CCTA has also been associated with higher utilization of medical therapies over functional testing when CAD is detected.^3,4^ However, the utility of CCTA for improving clinical endpoints in individuals ruled in for having obstructive CAD and undergoing PCI remains unexplored.

We conducted a retrospective analysis using the TriNetX U.S. Research Network—a federated, deidentified health data platform capturing real-world clinical data from various institutions within the United States. The primary objective was to evaluate whether individuals undergoing PCI within 1 year after CCTA experienced improved cardiovascular outcomes compared to those without a preceding CCTA (either proceeded directly to PCI or underwent functional evaluation).

Individuals aged ≥18 years who underwent PCI from January 2013 onwards were identified. To ensure a focus on *de novo* interventions and eliminate confounding from prior events, we excluded individuals with a history of or presenting acute coronary syndrome (unstable angina or MI), prior PCI, or coronary artery bypass grafting (CABG) that occurred at least 1 day before any instance of PCI to focus on new interventions. The exposure of interest was CCTA performed within 1 year prior to PCI. Patients were divided into two cohorts: those with a preceding CCTA (*n* = 4936) and those without (*n* = 80 370). ICD-10 and CPT coding were used for inclusion and exclusion criteria to ensure that the baseline presentations were similar in the two groups. Propensity score (PS) matching was conducted (1:1, nearest neighbour) to balance baseline covariates, including age, sex, hypertension, hyperlipidaemia, diabetes mellitus, tobacco use, obesity, and serum creatinine. The primary outcome was a composite of all-cause mortality, myocardial infarction (MI), or heart failure (HF) at 1 and 5 years. Secondary outcomes included the individual components of the composite. Kaplan-Meier survival curves and unadjusted hazard ratios (HRs) were used to assess for differences in the outcomes. *P* value < 0.05 was considered statistically significant. E-value was used in a sensitivity analysis, which quantifies the minimum strength of association that an unmeasured confounder would need with both the exposure and the outcome to fully explain away an observed effect. Higher E-values indicate more robust results, meaning the observed association is less likely to be due to unmeasured confounding. Typically, E-values >2 suggest moderately strong robustness, while values closer to 1 indicate that even weak confounders could negate the observed effect.^5^

After matching, 4936 patients remained in each group. Groups were well matched; in those with and without preceding CCTA age at index was 65.5 ± 10.5 vs. 65.5 ± 10.4 years old; male 66.7% vs. 69.8%; hypertension 79.9% vs. 80.0%; diabetes 33.1% vs. 33.0%; overweight/obesity 28.1% vs. 27.8%; hyperlipidaemia 73.7% vs. 73.6%; and smoking 16.8% vs. 16.5%.

At the 1-year follow-up, individuals with pre-PCI CCTA had significantly lower rates of the primary composite outcome compared to those without CCTA (476 events [9.6%] vs. 644 events [13.0%]; HR 0.74; 95% CI: 0.66–0.83) (E-value =2.12). At 5 years, the benefit persisted (741 events [16.1%] vs. 1150 events [23.7%]; HR 0.79; 95% CI: 0.72–0.86) (E-value =2.48). The benefit appeared to be driven primarily by lower rates of MI (137 events [2.8%] vs. 241 events [4.9]%; HR 0.57; 95% CI: 0.46–0.70 (E-value =3.02) at 1 year vs. 210 events [4.5%] vs. 420 events [9.1%]; HR 0.69; 95% CI: 0.59–0.81 (E-value =3.41) at 5 years) and death (64 events [1.3%] vs. 105 events [2.1%]; HR 0.62; 95% CI: 0.46–0.86 (E-value = 2.66) at 1 year vs. 148 events [3.2%] vs. 330 events [7.1%]; HR 0.63; 95% CI: 0.52–0.76 (E-value = 3.89) at 5 years). Although HF rates were not significantly different at 1 year (325 events [6.6%] vs. 381 events [7.7%]; HR 0.87; 95% CI: 0.75–1.01) (E-value = 1.80), a statistically significant reduction emerged at 5 years (449 events [10.8%] vs. 739 [26%]; HR 0.85; 95% CI: 0.76–0.95) (E-value = 2.33) (*Figure 1*).

**Figure 1:**
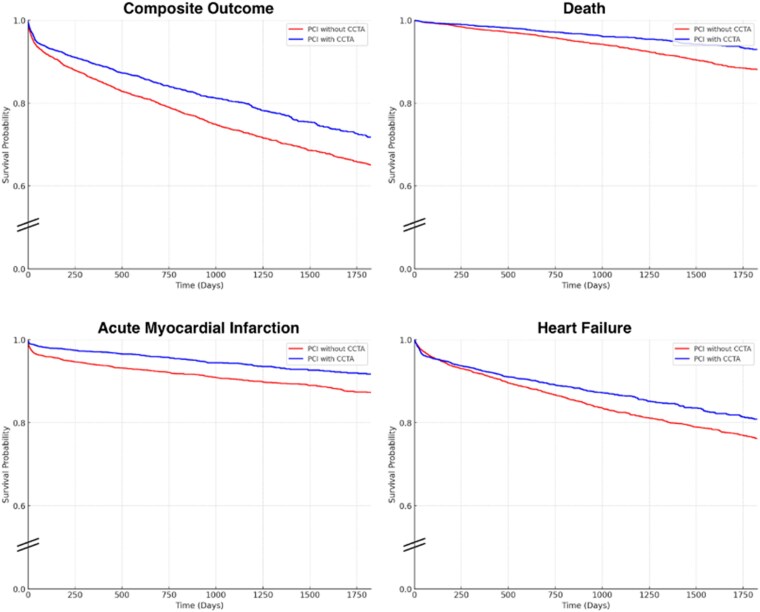
Kaplan-Meier survival analysis showing differences in 5-year outcomes between individuals undergoing percutaneous coronary intervention with a preceding coronary CTA angiogram (blue line) and those without a preceding coronary CTA (yellow line).

The results of the present analysis suggest that individuals who undergo a CCTA within a year of a PCI were associated with lower observed rates of adverse cardiovascular outcomes at both 1 and 5 years. While causality cannot be inferred due to the observational nature of the study, this association could be influenced by the detailed anatomical characterization provided by CCTA, which might assist in pre-procedural revascularization planning or guide medical optimization, although this could not be confirmed in our dataset.

Several mechanisms might help explain the observed association. First, CCTA allows for noninvasive, high-resolution visualization of coronary anatomy, aiding in the identification of atherosclerotic plaque morphology, vessel tortuosity, and lesion length, all of which influence PCI planning. Second, the use of CCTA may facilitate better selection of patients for invasive angiography, potentially reducing the likelihood of incomplete or inappropriate revascularization. Last, the additional time afforded by the pre-procedural workup may coincide with opportunities for the optimization of risk factors and pharmacologic therapy, although this could not be measured in this analysis.

This is one of the largest real-world analyses to evaluate pre-PCI CCTA, using a multi-institutional dataset with robust statistical adjustment. The persistence of the association over 5 years highlights a potentially meaningful signal that warrants further investigation.

Nonetheless, important limitations warrant consideration. The retrospective design risks residual confounding despite matching. Data lacked certain information, such as procedural details and CCTA indications, which may introduce selection bias. Importantly, the TriNetX research platform, while offering access to a large, diverse patient population across multiple healthcare institutions, does not permit access to highly granular clinical data. This includes variables such as lesion morphology, left ventricular ejection fraction, stent type and size, completeness of revascularization, procedural strategy, or coronary segment involvement. As such, several clinically relevant factors that could influence outcomes—particularly myocardial infarction and heart failure—could not be adjusted for or evaluated. These limitations reflect the inherent constraints of using real-world, deidentified federated datasets. Furthermore, cause-specific mortality and certain cardiovascular endpoints were unavailable. The absence of randomization limits causal inference.

Additionally, the long inclusion period (2013–2025) may introduce secular trends related to evolving PCI practices and medical therapy. While this limitation cannot be fully mitigated, it reflects real-world clinical evolution over time across diverse care settings. The observed reduction in HF at 5 years should be interpreted cautiously, as it may reflect competing risks or differential survival rather than a direct effect, particularly in the absence of baseline left ventricular function data. Throughout, we emphasize that the observed differences represent associations rather than causal effects, given the retrospective design and absence of key clinical variables. Finally, information on the use of photon-counting CT technology and detailed scanner-level parameters, including average spatial CT resolution, is not available within the TriNetX platform and therefore could not be assessed in this study.

In conclusion, this real-world retrospective analysis suggests that undergoing CCTA within 1 year prior to PCI is associated with lower rates of adverse cardiovascular outcomes at both 1 and 5 years. However, given the observational nature of the study and the absence of several important clinical variables, these findings should be interpreted as hypothesis-generating rather than conclusive. The results highlight the potential value of anatomical imaging in the pre-PCI setting but require validation in prospective studies that can more comprehensively account for clinical, procedural, and pharmacologic variables.

## Data Availability

The data that support the findings of this study were obtained from the TriNetX U.S. Research Network, a federated, real-world data platform that aggregates de-identified clinical information from multiple healthcare organizations. Due to licensing and data use agreements, individual-level data from TriNetX cannot be shared publicly. Access to the TriNetX platform is available to participating institutions via a commercial license. Researchers interested in accessing similar data may contact TriNetX at (https://www.trinetx.com) to inquire about institutional access.

## Lead author biography

Khalid Sawalha, MD, is a cardiology fellow at the University of Arkansas for Medical Sciences with clinical and research interests in preventive cardiology, cardiometabolic health, coronary artery disease, cardiovascular imaging, and interventional cardiology, including mechanical circulatory support and critical care. He completed advanced training in cardiometabolic medicine at the University of Missouri-Kansas City and plans to pursue interventional cardiology fellowship training following completion of his general cardiology fellowship. Dr. Sawalha has authored over 60 peer-reviewed publications.
